# Association between socioeconomic status and patient-reported outcome at 1 year after shoulder arthroplasty for osteoarthritis or cuff-tear arthropathy: a nationwide cohort study of 2,292 arthroplasties

**DOI:** 10.2340/17453674.2024.42700

**Published:** 2025-01-09

**Authors:** Marie L JENSEN, Epaminondas M VALSAMIS, Alexander S MADRID, Bo S OLSEN, Jeppe V RASMUSSEN

**Affiliations:** 1 Copenhagen University Hospital, Herlev and Gentofte, Denmark; 2 Botnar Research Centre, Nuffield Department of Orthopaedics, Rheumatology and Musculoskeletal Sciences, University of Oxford, Oxford, UK

## Abstract

**Purpose:**

We aimed to evaluate the association between socioeconomic factors and patient-reported Western Ontario Osteoarthritis of the Shoulder (WOOS) index at 1 year after hemiarthroplasty, reverse, or anatomical total shoulder arthroplasty for osteoarthritis or cuff-tear arthropathy.

**Methods:**

Eligible patients were identified using linked national data from the Danish Shoulder Arthroplasty Registry and Statistics Denmark between April 2012 and April 2019. Univariable and multivariable linear regression was used to identify the association between socioeconomic factors and the WOOS index at 1 year following primary shoulder arthroplasty adjusted for age, sex, underlying diagnosis, implant design, and comorbidities. We examined socioeconomic factors including employment status, marital status, education, and income. Estimates were provided with 95% confidence intervals (CI).

**Results:**

2,292 patients were identified with a mean WOOS index of 76 (standard deviation 24). In the adjusted analysis, unemployed patients had a significantly lower WOOS index compared with patients with low-level jobs (14, CI 7.0–21), patients with high-level jobs (19, CI 12–25), and retired patients (14, CI 8.3–21). Low education level was associated with a lower WOOS index compared with medium education (4.8, CI 2.6–7.0) and high education level (7.7, CI 5.0–10). There was no association between WOOS index and income or marital status.

**Conclusion:**

Unemployment and low education level were associated with worse WOOS index 1 year after shoulder arthroplasty for osteoarthritis or cuff-tear arthropathy. This highlights a potential inequity in patient-reported outcomes after shoulder arthroplasty.

Shoulder arthroplasty remains the main treatment option for end-stage glenohumeral osteoarthritis (OA) and cuff-tear arthropathy (CTA). The patient-reported Western Ontario Osteoarthritis of the Shoulder (WOOS) index has improved over time for patients treated with shoulder arthroplasty for OA, likely in part due to increased use of reverse and anatomical total shoulder arthroplasties [1]. While most patients having a shoulder arthroplasty report improvement in pain and function after surgery, some patients have poorer function, suffer from medical complications, or need revision surgery [1,2]. Identifying factors that are associated with poor outcomes is important to facilitate patient–doctor counselling, to select the best treatment for each individual patient, and for the efficient targeting of healthcare resources to improve patient care.

Socioeconomic factors have been shown to be associated with clinical and patient-reported outcomes after surgery, and more deprived groups have a higher risk of complications [3,4].

A study performed on Danish patients reported an increased incidence of revision surgery and death after hip arthroplasty in patients with low income, low liquid assets, and who were living alone [5]. While revision surgery and complication rates are important outcomes, patient-reported outcome measures (PROMs) provide an important indicator of patient-centered treatment efficacy [6]. PROMs may also capture those patients with poor function after surgery, but who do not undergo revision surgery. This could be patients with severe comorbidity, if the revision arthroplasty is expected to be technically demanding etc. To date, there is no published literature describing the association between socioeconomic factors and results evaluated of WOOS index following shoulder arthroplasties.

We aimed to evaluate the association between socioeconomic factors and the WOOS index following elective primary shoulder arthroplasty for OA and CTA.

## Method

This was a population-based, nationwide cohort study using linked data from the Danish Shoulder Arthroplasty Registry (DSR), Danish National Patient Registry (DNPR), and Statistics Denmark (STD). We report this study according to the Reporting of studies Conducted using Observational Routinely-collected Data (RECORD) guidelines.

### Data source

In Denmark, all citizens are registered with a unique civil registration (CPR) number received at immigration or birth. The Danish healthcare system is fundamentally tax-funded and free for all at the point of demand. Healthcare providers are reimbursed for the services they provide by reporting data, linked to the CPR number for all treated patients, to the DNPR. A few shoulder arthroplasties were performed at private clinics, mostly funded by insurance companies from the public healthcare system.

Since 2006, reporting to the DSR has been mandatory for all public hospitals and private clinics in Denmark. It contains surgical and patient-related data reported by the surgeon at the time of the surgery [7]. The completeness of reporting has been above 90% each year since 2007, and the overall completeness is 94% [7]. Linkage of data is done through the unique CPR number. The WOOS index questionnaire at 1 year after surgery was distributed via postal mail [8].

STD provided data regarding socioeconomic status based on the following variables: highest formal level of education, income, current employment status, and marital status.

### Population

We included patients over 18 years of age having surgery for OA and/or CTA. The exclusion criteria were other diagnoses (avascular necrosis, fracture sequelae, acute fracture, inflammatory arthritis, malignancy, other diagnoses), students (as there was only 1) and missing key values (diagnosis, WOOS index, education, arthroplasty type). Charlson comorbidity index score was retrieved from the DSR.

**Figure 1:**
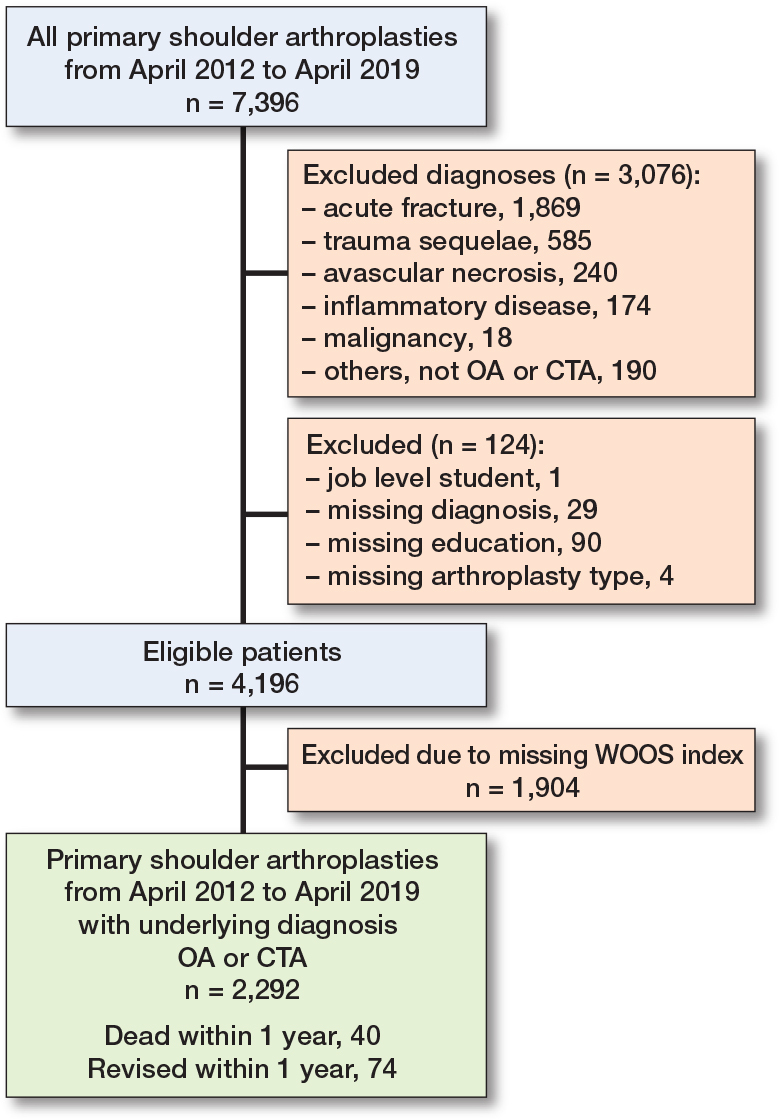
Patient flowchart. CTA = cuff-tear arthropathy. OA = osteoarthritis. WOOS = Western Ontario Osteoarthritis of the Shoulder.

After application of the eligibility criteria, 2,292 primary shoulder arthroplasties remained (Figure 1).

### Socioeconomic status

Employment status at the time of surgery was categorized into 5 groups: unemployed, low-level job, high-level job, retired, and others.

Patients were considered unemployed if they had no job for at least 6 months prior to surgery or were solely dependent on social care. A low-level job was defined as employment that did not require any formal education, including primary school. A high-level job was defined as being a manager, self-employed (with any number of employees), or having a job that requires an education (e.g., artisans, doctors, schoolteachers, engineers, etc.). Retirement included patients who were retired for any reason, including due to age (in Denmark the current age of retirement is 67 years) or for physical/psychological reasons, or patients who relied on a private pension or funding.

Education level was derived from the highest formal level of education at the time of primary surgery and was categorized into 3 groups. Marital status was categorized into the following categories: no registered partnership, widowed, registered partnership, and terminated partnership. Income represented the total available income for the year of the primary surgery.

Variable specifications are available as Supplementary data on the on the article page.

### Outcome

The WOOS index is a disease-specific questionnaire aimed at and validated for evaluating patients with osteoarthritis of the glenohumeral joint. It consists of 19 questions within the 4 domains of pain, mobility, lifestyle, and emotions [9]. Patients provide responses on a visual scale from 0 to 100 for each question with a total score between 0 and 1,900, with 1,900 representing the worst possible outcome [9]. For easier interpretation and presentation, the WOOS index was converted to a score ranging from 0–100 with 100 being the best score, in keeping with previous studies [1,10,11].

In the case of revision or death within 1 year of surgery, the WOOS questionnaire was not sent to the patient. In the case of revision later than 1 year after surgery, the WOOS index was recorded as usual and included in the analysis.

There is no published minimal clinically important difference (MCID) for the specific cohort of patients included in this study, but the MCID identified in a study that included only anatomical total shoulder arthroplasties for osteoarthritis was 12.3 at 1 year after surgery using the converted WOOS index [11]. The MCID is used to determine whether a difference is regarded important but does not incorporate any information on, e.g., burden of society, costs, risks etc. regarding the magnitude of the difference [12]. We therefore decided to use the MCID value as guidance as to whether the results are clinically important [11].

### Statistics

**Figure 2:**
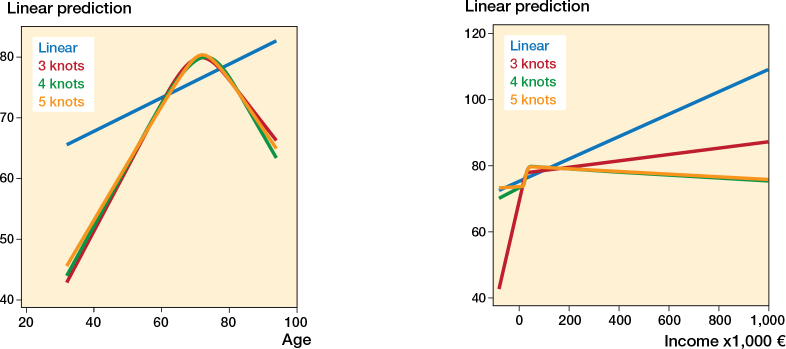
Evaluation of non-linearity for the continuous variables age (left panel) and income (right panel). Age: Linear: AIC 21067.55 BIC 21079.03 3 knots: AIC 21003.67 BIC 21020.88 4 knots: AIC 21005.28 BIC 21028.22 5 knots: AIC 21006.97 BIC 21035.65 Income: Linear: AIC 21087.98 BIC 21099.46 3 knots: AIC 21081.16 BIC 21098.37 4 knots: AIC 21078.04 BIC 21100.99 5 knots: AIC 21079.94 BIC 21108.62

Baseline patient demographic data was reported using descriptive statistics. Univariable and multivariable linear regression models were used to identify the associations between socioeconomic factors and the WOOS index. Variables included in the multivariable linear model were chosen a priori following discussion with clinical experts and review of the literature [2,3,13]. Within the limitations of the available data, we included sex, age, arthroplasty type, underlying diagnosis, Charlson comorbidity index group, income, marital status, employment status, and education level. Age and income were used as continuous variables, conforming to the guidelines by Collins et al. [14]. Non-linearity of continuous outcomes was evaluated using restricted cubic splines, and the most parsimonious specification was chosen based on graphical inspection, the Akaike information criterion (AIC), and the Bayesian information criterion (BIC). Age and income were modelled using restricted cubic splines with 3 knots (Figure 2, see Appendix). Differences between the socioeconomic groups decrease by the multivariable analysis, and measurement uncertainties remain approximately equal. Interaction analyses were performed on the following suspected variables, chosen a priori, using likelihood ratio tests: employment status and income, employment status and education, as well as income and education. P < 0.05 was considered statistically significant [15]. Estimates were provided with 95% confidence intervals (CI). Stata MP statistical software (Version 18.0; StatCorp LLC, College Station, TX, USA) was used for all analyses.

### Missing data

Surgical indication was missing in 29 (0.4%) patients, education level was missing in 90 (1.2%) patients, and arthroplasty type was missing in 4 (0.1%) patients. These records were excluded, and a complete case analysis undertaken. Where any question was not completed within each WOOS index questionnaire (n = 1,904, representing 45%), the record was considered void and excluded. Multiple imputation was not undertaken for missing WOOS indices as those scores are expected to be missing not at random (MNAR), and previous research has found an association of increased PROM missingness in patients with poorer outcomes and increased revision surgery rates, making it unlikely that any imputation would be accurate and reliable [16]. There were no major differences in baseline data for responders and non-responders (Table 1, see Appendix). It is noteworthy that the group of non-responders had a larger proportion of unemployment (5.6% vs 3.1%) and a higher proportion of low education level (39% vs 34%).

**Table 1:** Demographics for patients with missing WOOS index vs non-missing. Values are count (%) unless otherwise specified

Variable	Non-missing WOOS index	Missing WOOS index
Age, mean (SD)	71 (8.9)	70 (10)
Sex		
Female	1,373 (60)	1,210 (64)
Male	919 (40)	694 (36)
Underlying diagnosis		
Cuff -tear arthropathy	723 (32)	673 (35)
Osteoarthrosis	1,569 (68)	1,231 (65)
Arthroplasty design		
Anatomical total shoulder arthroplasty	970 (42)	807 (42)
Reverse shoulder arthroplasty	886 (39)	715 (38)
Hemi-arthroplasty	436 (19)	382 (20)
Employment status		
Unemployed	72 (3.1)	106 (5.6)
Low-level job	104 (4.5)	81 (4.2)
High level job	156 (6.8)	99 (5.2)
Retired	1,885 (82)	1,556 (82)
Others	75 (3.3)	62 (3.3)
Income, mean (SD), € per year	27,000 (27,192)	25,238 (18,891)
Education level		
Low	789 (34)	740 (39)
Medium	1,004 (44)	795 (42)
High	499 (22)	369 (19)
Marital status		
No registered partnership	596 (26)	391 (21)
Widow	344 (15)	325 (17)
Registered partnership	1,096 (48)	878 (46)
Terminated partnership	256 (11)	310 (16)
Charlson Comorbidity Index		
0	1,232 (54)	980 (51)
1	447 (20)	401 (21)
≥2	613 (27)	523 (27)

### Ethics, funding, data sharing, use of AI, and disclosures

The project was approved by the Danish Data Protection Agency (approval number P-2022-898). Data for this study was stored on an online server hosted by STD. Data access requires certification by STD. The study was funded by the Department of Orthopaedic Surgery at Gentofte Hospital. EMV was funded by a National Institute for Health and Care Research (NIHR) Doctoral Research Fellowship. The funders had no role in the study design, data collection, management, analysis, interpretation of data, decision to submit, or writing of the manuscript.

BSO has received a study grant from Depuy Synthes as investigational support for a randomized controlled trial of the Delta Xtend arthroplasty, which author MLJ performs. BSO has given lectures sfor Depuy Synthes and Swemac. BSO and JVR are members of the board of the DSR. JVR is treasurer of the Danish Orthopedic Society. EMV and ASM declare no conflicts of interest. Complete disclosure of interest forms according to ICMJE are available on the article page, doi: 10.2340/17453674.2024.42700

## Results

7,396 primary shoulder arthroplasties were reported to the DSR from April 2012 until April 2019. In the study period, there were 4,196 primary shoulder arthroplasties that met the inclusion criteria. Of these, 74 patients underwent revision and 40 died within 1 year after surgery and therefore did not receive a WOOS questionnaire. Thus, 4,082 received a WOOS questionnaire among whom 1,790 (44%) patients did not return a complete WOOS questionnaire. There were 136 arthroplasties that were revised later than 1 year after surgery. After application of the eligibility criteria, 2,292 primary shoulder arthroplasties remained (Figure 1). The mean age was 71 years. The mean income was €27,000 for the year of surgery and income was retrieved in 1,885 patients (82%). Most patients (44%) had a medium level of education, 34% had a low level of education, and 22% had a high level of education. Patients with a high-level job had the highest WOOS index while those who were unemployed had the lowest (Tables 2 and 3).

**Table 2:** Demographics for primary shoulder arthroplasties with the diagnoses cuff-tear arthropathy or osteoarthritis

Variable	Primary surgery n (%)	WOOS index mean (SD)
Age, mean (SD)	71 (8.9)	
Sex		
Female	1,373 (60)	76 (24)
Male	919 (40)	76 (24)
Underlying diagnosis		
Cuff-tear arthropathy	723 (32)	70 (25)
Osteoarthritis	1,569 (68)	79 (23)
Arthroplasty design		
Anatomical total shoulder arthroplasty	970 (42)	82 (22)
Reverse shoulder arthroplasty	886 (39)	73 (24)
Hemi-arthroplasty	436 (19)	70 (26)
Employment status		
Unemployed	72 (3.1)	54 (29)
Low-level job	104 (4.5)	72 (25)
High-level job	156 (6.8)	79 (21)
Retired	1,885 (82)	77 (24)
Others	75 (3.3)	74 (25)
Income, mean (SD), € per year	27,000 (27,192)	
Education level		
Low	789 (34)	71 (27)
Medium	1,004 (44)	77 (23)
High	499 (22)	82 (20)
Marital status		
No registered partnership	596 (26)	73 (25)
Widow	344 (15)	77 (22)
Registered partnership	1,096 (48)	78 (24)
Terminated partnership	256 (11)	75 (26)
Charlson Comorbidity Index		
0	1,232 (54)	78 (23)
1	447 (19)	74 (25)
≥ 2	613 (27)	74 (25)

**Table 3:** Univariable and multivariable analysis for the WOOS index in percentage points 1 year after primary surgery

Variable	Univariable coefficient (CI)	P value	Multivariable coefficient (CI)	P value
Age ^a^				
Spline 1	1.0 (0.8 to 1.3)	< 0.001	1.0 (0.7 to 1.3)	< 0.001
Spline 2	–1.0 (–1.2 to –0.7)	< 0.001	–0.7 (–1.0 to –0.5)	< 0.001
Sex				
Female	Ref		Ref	
Male	–0.3 (–2.3 to 1.7)	0.8	2.6 (0.5 to 4.7)	0.02
Underlying diagnosis				
Osteoarthritis	Ref		Ref	
Cuff-tear arthropathy	–8.5 (–11 to –6.4)	< 0.001	–6.8 (–9.6 to –4.1)	< 0.001
Arthroplasty design				
Anatomical total shoulder	Ref		Ref	
Reverse shoulder	–10 (–12 to –7.3)	< 0.001	–5.2 (–8.1 to –2.4)	< 0.001
Hemi-arthroplasty	–12 (–15 to –9.6)	< 0.001	–9.4 (–12 to –6.7)	< 0.001
Employment status				
Unemployed	Ref		Ref	
Low-level job	19 (11 to 26)	< 0.001	14 (7.0 to 21)	< 0.001
High-level job	26 (19 to 32)	< 0.001	19 (12 to 25)	< 0.001
Retired	23 (18 to 29)	< 0.001	14 (8.3 to 21)	< 0.001
Others	20 (13 to 28)	< 0.001	15 (7.3 to 22)	< 0.001
Education level				
Low	Ref		Ref	
Medium	6.1 (4 to 8)	< 0.001	4.8 (2.6 to 7.0)	< 0.001
High	10 (8 to 13)	< 0.001	7.7 (5.0 to 10)	< 0.001
Income ^a^				
Spline 1	0.3 (0.1 to 0.5)	< 0.001	0.2 (0.0 to 0.4)	0.05
Spline 2	–0.3 (0.5 to –0.1)	0.003	–0.2 (–0.4 to 0.0)	0.05
Marital status				
No registered partnership	Ref		Ref	
Widow	4.1 (0.9 to 7.3)	0.01	1.1 (–2.3 to 4.4)	0.5
Registered partnership	4.4 (2.0 to 6.8)	< 0.001	0.7 (–1.8 to 3.1)	0.7
Terminated partnership	1.6 (–1.9 to 5.1)	0.4	–0.8 (–4.2 to 2.7)	0.6
Charlson Comorbidity Index				
0	Ref		Ref	
1	–3.7 (–6.3 to –1.1)	0.01	–3.9 (–6.3 to –1.4)	0.002
≥ 2	–4.3 (–6.6 to –2.0)	< 0.001	–3.9 (–6.2 to –1.7)	0.001

a Restricted cubic spline.

Knot locations age: 58, 72, 81.

Knot locations income: 13.9, 22.8, 41.8.

In the multivariable analysis, unemployment was associated with a lower WOOS index compared with patients with low-level jobs (14, CI 7.0–21), patients with high-level jobs (19, CI 12–25), and retired patients (14, CI 8.3–21). Low education level was associated with a lower WOOS index compared with medium education (4.8, CI 2.6–7.0) and high education level (7.7, CI 5.0–10). There is uncertainty as to whether the effect sizes for these categories are clinically important as there is no certain estimate of clinical relevance compatible with this study [17,18]. Income and marital status were not statistically significantly associated with the WOOS index.

The proportion of revised arthroplasties in socioeconomic groups showed a small difference between the socioeconomic groups (Table 4).

**Table 4:** Number of patients revised from total population of 4,196 patients, n (% of total of subgroup ^a^)

Variable	Revised		total
	within 1st year	after 1 year	
Number revised	74 (1.8)	136 (3.2)	210 (5.0)
Employment status			
Unemployed	4 (2.2)	13 (7.3)	17 (9.6)
Low-level job	4 (2.2)	15 (8.1)	19 (10)
High-level job	5 (2.0)	11 (4.3)	16 (6.3)
Retired	59 (1.7)	93 (2.7)	152 (4.4)
Others	2 (1.5)	4 (2.9)	6 (4.4)
Education level			
Low	37 (2.4)	54 (3.5)	91 (6.0)
Medium	24 (1.3)	63 (3.5)	87 (4.8)
High	13 (1.5)	19 (2.3)	32 (3.7)
Income			
< €25,000	48 (1.8)	84 (3.2)	132 (5.0)
> €25,000	26 (1.7)	52 (3.3)	78 (5.0)
Marital status (registered partnership)			
No	24 (2.4)	62 (6.3)	86 (8.7)
Widow	8 (1.2)	9 (1.3)	17 (2.5)
Yes	32 (1.6)	51 (2.6)	83 (4.2)
Terminated	10 (1.8)	14 (2.5)	24 (4.3)

a Percentages are reported per total in subgroup, e.g., retired revised within first year/total of retired.

## Discussion

We aimed to evaluate the association between socioeconomic factors and patient-reported Western Ontario Osteoarthritis of the Shoulder (WOOS) index at 1 year after hemiarthroplasty, reverse, or anatomical total shoulder arthroplasty for osteoarthritis or cuff-tear arthropathy. We found a lower WOOS index in patients who were unemployed and had a low education level, though the clinical importance remains uncertain. No statistically significant association was found for income and marital status.

To our knowledge, this is the first study showing an association between employment status and the WOOS index after shoulder arthroplasty. Previous studies have investigated the association between socioeconomic status and patient outcomes after different types of surgery. They have found low income, living alone, and low liquid assets to be the most significant factors for serious adverse events after hip arthroplasty [5], and income and liquid assets are known to be associated with job status [19]. We did not find income to be statistically significantly associated with the WOOS index. A population-based study from Sweden and a psychological theoretical model from Duke University imply that unemployment has a negative effect on health outcomes [20,21], comparable to this study. A previous study of patients with hip fractures found that access to and use of physiotherapy varied with employment status, income, and education [22]. It is not known whether there are similar associations for shoulder arthroplasty patients.

While the confidence intervals of the employment coefficients included the MCID value, the point estimates were higher than the MCID in all cases. Moreover, the MCID value used in this study was developed using an anchor-based method, which is relatively conservative, and other studies have identified lower values to be clinically important [11,23]. Other parameters, like personal health literacy, may also influence what a person or a group of persons perceives as being clinically important. Due to the absence of alternatives, the MCID was used as guidance for evaluation of clinical importance [11].

Income was not associated with the WOOS index, which suggests that the underlying explanation could be a psychological sentiment of purpose. Psychological studies have found that being employed increases reported self-satisfaction [21]. This could explain the association we identified between unemployment and the WOOS index, some questions of which pertain to emotion, especially if surgery does not enable the patient to return to work. Additionally, unemployed patients might experience more pain or have reduced benefit from rehabilitation [21]. There is a certain possibility that the socioeconomic variables are interrelated. This is illustrated in the analysis, as the differences in the adjusted analysis are decreased in relation to the unadjusted analysis. However, suspected interactions were defined prior to analysis and were not found to be significant.

Any revision will probably influence the outcome. However, if patients are revised within the first year after the surgery, they will not receive a WOOS index questionnaire before their revision, but 1 year after the revision. In this study, revision surgeries were not included, causing these patients to be reported as having missing WOOS indices. Some patients have a low WOOS index at 1 year after surgery, without being revised, which can be due to different factors such as comorbidities, technical challenges, or the patient’s wishes. Table 3 shows the distribution of revision surgeries within the first year and later, as well as the distribution between socioeconomic groups. As our study concerns PROMs, we choose not to perform a time-dependent analysis, but it would be interesting for a future study.

### Strengths

The main strength of this study lies in the use of a large, national, linked database capturing patients from all socioeconomic backgrounds and levels of comorbidity. The linkage of the different databases enables comprehensive confounding adjustment, as a number of relevant variables collected by different databases were available. The large sample size for this type of study enables a more accurate estimate of the effect size of different variables. Considering the results in reference to an MCID value, they improve aids to determining the clinical relevance of the findings.

### Limitations

First, it is possible that there might be reverse causality bias if patient employment is associated with the severity of their shoulder condition, meaning that some patients may have become unemployed due to the pain and reduced function from their glenohumeral osteoarthritis.

Second, the preoperative WOOS index is not routinely collected in the DSR, which is a major limitation of this study. It is important to keep in mind that any systematic differences in pre-operative WOOS index could influence the results and thereby the conclusion.

Third, the missingness of the WOOS index was relatively high (45%), though imputation was deemed inappropriate due to concerns regarding missing data bias.

If there were any systematic differences in responders and non-responders, the results might be skewed due to response bias. A previous study investigated differences between responders and non-responders to the WOOS questionnaire in the Danish population and did not find demographic differences or significant differences in overall WOOS index [10]. However, if non-responders were included, thereby avoiding response bias, generally differences in socioeconomic factors would be greater.

### Conclusion

We found that unemployment and low education level were associated with worse WOOS index 1 year after shoulder arthroplasty for osteoarthritis or cuff-tear arthropathy.

*In perspective,* the findings highlight a potential inequity in patient-reported outcomes after shoulder arthroplasty. Thorough preoperative information given to the patient, shared decision-making, and optimized individual planned postoperative processes may increase patient-reported outcomes in the future.

### Supplementary data

Socioeconomic variable specifications are available as Supplementary data on the article page, doi: 10.2340/17453674.2024.42700

## Supplementary Material



## Notes

All authors contributed to the study design. The analysis and interpretation of results were performed by MLJ, EMV, ASM, BSO, and JVR. All authors meet the co-author criteria and no others meet the criteria. All authors contributed to writing the manuscript and have approved the final manuscript.

The authors would like to thank Mark Alexander Henney, MSc, who was consulted in the coding of data preparation in R.

Handling co-editor: Taco Gosens

*Acta* thanks Joshua M. Bonsel and Jan-Erik Gjertsen for help with peer review of this manuscript.
