# Supplementary material for: Association between socioeconomic status and patient-reported outcome at 1 year after shoulder arthroplasty for osteoarthritis or cuff-tear arthropathy: a nationwide cohort study of 2,292 arthroplasties
Source: Acta Orthop. 2025 Jan 9;96:45–51. doi: 10.2340/17453674.2024.42700 (PMC11718085; doi:10.2340/17453674.2024.42700)
Supplement: Supplementary file 1 [file ActaO-96-42700-s1.pdf]

Supplementary data. Socioeconomic variable specifications

STD categorization	Categorization for this study
HFAUDD*<br>400 categories describing specific education, though divided into the following categories: no education, primary school, high school, vocational education, short higher education, medium–long higher education, long higher education, others (different education with around 3.5 years of development of special abilities).	Education level:<br>Low:<br>• Primary school<br>• No education<br><br>Medium:<br>• High school<br>• Vocational education<br>• Short higher education<br>• Others<br><br>High:<br>• Medium–long higher education<br>• Long higher education
DISPINDK1*<br>Calculated income after taxes, interest expenses, pension contribution, and other payments to the state In DKK per year	Income<br>• Continuous variable per €1,000
SOCIO13*<br>22 categories describing if self-employed with number of employees, manager position, employee with requirement for basic, intermediate or high qualifications, unemployed, public welfare, retired, student, child, and others	Current employment status<br>High-level job:<br>• Self-employed<br>• Manager position<br>• High qualifications required<br>• Intermediate qualifications required<br><br>Low-level job:<br>• Basic qualifications required<br><br>Unemployed:<br>• Unemployed at least half the year<br>• Public welfare<br><br>Retired:<br>• Early retirement<br>• Retirement<br><br>Students and children<br>• Student<br>• Child<br><br>Others<br>• Job that required specific qualifications, but not education
CIVST [a]	Marital status:<br>Widow

Divided into the following categories: widowed, divorced, married, longest living partner, terminated partnership, registered partnership, no registered partnership	• Widowed<br>• Longest living partner<br>Terminated partnership<br>• Terminated partnership<br>• Divorced<br>Registered partnership<br>• Married<br>• Registered partnership<br>No registered partnership<br>• Single<br>• No registered partnership

[a] Original name of variables listed on www.dst.dk, where more detailed descriptions for each variable can be found.
